# Supplementary material for: Disentangling the causes of temporal variation in the opportunity for sexual selection
Source: Nat Commun. 2023 Feb 22;14:1006. doi: 10.1038/s41467-023-36536-7 (PMC9947164; doi:10.1038/s41467-023-36536-7)
Supplement: Supplementary file 1 — Supplementary information [file 41467_2023_36536_MOESM1_ESM.pdf]

Supplementary information for: Disentangling the causes of temporal variation in the opportunity for sexual selection

Rômulo Carleial[1,2*], Tommaso Pizzari[1], David S. Richardson[3], Grant C. McDonald[4*]

[1]Department of Zoology, Edward Grey Institute, University of Oxford, Oxford, OX1 3SZ, United Kingdom

[2]Science Directorate, Royal Botanic Gardens, Kew, Richmond TW9 3AE, United Kingdom

[3]School of Biological Sciences, University of East Anglia, Norwich, United Kingdom

[4]Department of Ecology, University of Veterinary Medicine Budapest, Budapest 1077, Hungary

*Corresponding authors: Rômulo Carleial email: r.carleial@kew.org; Grant C. McDonald email: grant.mcdonald@univet.hu

Supplementary Information: Supplementary Table 1; Supplementary Figures 1–2

Supplementary Table 1 - Summary information for empirical studies used in analyses of the opportunity for precopulatory sexual selection on mating success (I_M). Studies are sorted by Taxon.

Study	Species	Taxon	Mating system	Type	Description	Comments
Sih et al. 2017[1]	*Aquarius remigis*	Insects	Polygynandry	Lab	24 groups containing 3 males and 3 females each were observed for 6 consecutive days. Groups were then mixed into larger groups of 12 individuals of each sex, which were then observed for 3 days. Finally, large groups were split once again into smaller groups of 3 individuals per sex and observed for an extra 6 days.	To eliminate the effects of group size and social experience, we only used the first 6 days of the experiment when the 24 groups were isolated.
Turnell & Shaw 2015[2]	*Laupala cerasina*	Insects	Polygynandry	Lab	A group comprising 20 males and 20 females were observed over 6 weeks. A total of 6 older females were substituted by 6 young females on the 5th day of the experiment. A total of 5 males and a single female died over the experiment and were replaced with new	No replicates. We could not identify which individuals were replaced over the experiment, so we assumed all unique individuals in the dataset were

					individuals. Only 15 males were used in the final analysis.	present throughout the experiment.
Boinski 1987[3]	*Saimiri oerstedi*	Mammals	Polygyny	Field	A single troupe of squirrel monkeys (ranging from 38-45 individuals) was observed for 17 to 21 days per month for 11 months. 10 males and 16 females were reproductively mature during observations.	No replicates. We assumed all individuals were present throughout the observation period. Only sexually mature individuals were included. Matings with unknown individuals were not included in the calculation of mating success.
Oklander et al. 2014[4]	*Alouatta caraya*	Mammals	Polygynandry	Field	Data comprised 2 multi-male multi-female groups were observed on 5 separated sampling periods lasting 2 weeks each. One group had 3 adult males and 4 adult females, whereas the other had 3 adult males and 2 adult females. Extra-group matings occurred in 2 sampling seasons.	We considered each sampling period as a replicate group. Although both groups differed in sex ratio and group size, they were used together in the analyses. In the two sampling periods in which extra-group matings occurred, both groups were treated as one larger group.
Pröhl & Hödl 1999[5]	*Dendrobates pumilio*	Amphibia	Sequential polygamy	Field	Individuals were observed in the field for 143 consecutive days. 9 males and 11 females mated over the sampling period.	No replicates. Matings with unknown individuals were not included in the

						calculation of mating success.
Gill et al. 2020[6]	*Corvus modenula*	Birds	Social monogamy	Lab	8 nest boxes were sampled over a period of 22 days using video recordings. 18 individuals mated and were properly identified.	No replicates. Matings with unknown individuals were not included in the calculation of mating success.
McDonald et al. 2017[7]	*Gallus gallus*	Birds	Polygynandry	Lab	Freely-mating groups (n=20) containing 10 males and 12 females each. Groups were observed 6h a day for 10 days. Eggs were collected at the end of each day. Parentage analyses were performed for each fertilised egg.	Some males and females were reused across groups.

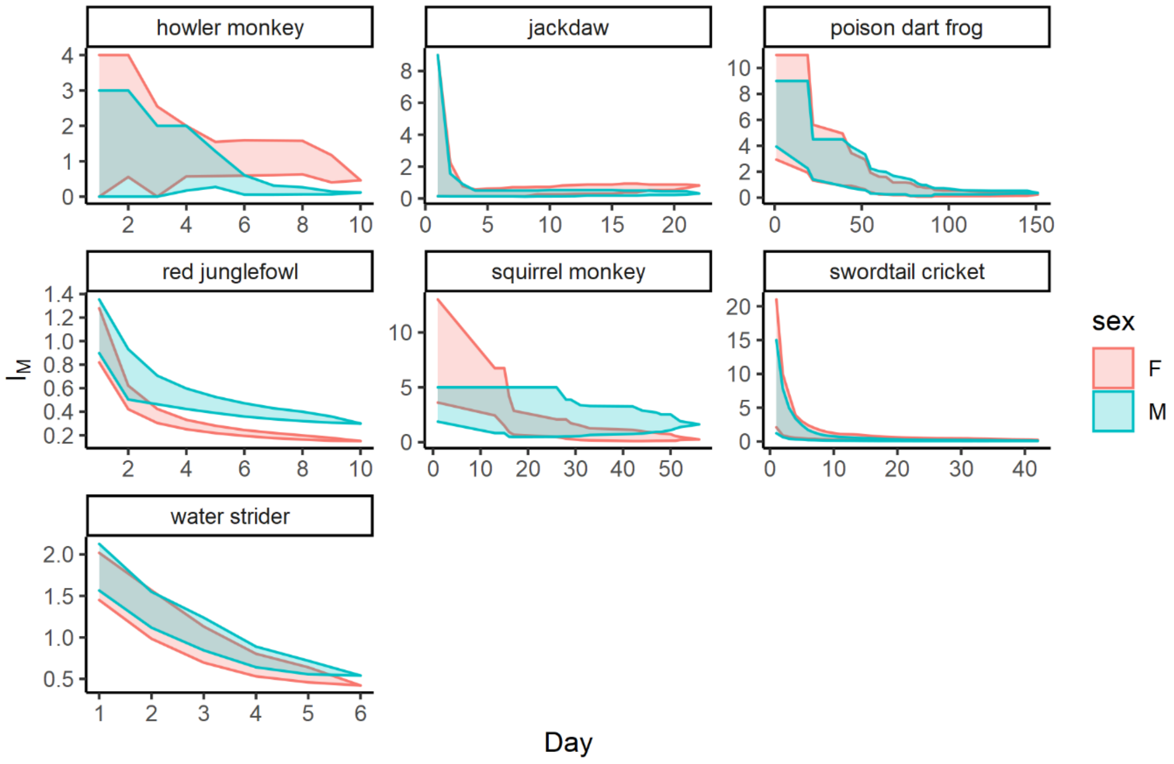

Supplementary Figure 1 – Impact of reshuffling day order on temporal patterns in the opportunity for pre-copulatory sexual selection (I_M) across multiple animal species. Species include Hawaiian swordtail crickets[2] (*Laupala cerasina*), strawberry poison-dart frogs[5] (*Dendrobates pumilio*) *n* groups = 1, howler monkeys[4] (*Alouatta caraya*) *n* groups = 6, jackdaws[6] (*Corvus monedula*) *n* groups = 1, red junglefowl[7-10] (*Gallus gallus*) *n* groups = 20, squirrel monkeys[3] (*Saimiri oerstedi*) *n* groups = 1, and water striders[1] (*Aquarius remegis*) *n* groups = 40. Results show the 95% range of 1000 simulations in which days were reshuffled arbitrarily. Female (F) patterns are colour coded in red and male patterns (M) in green.

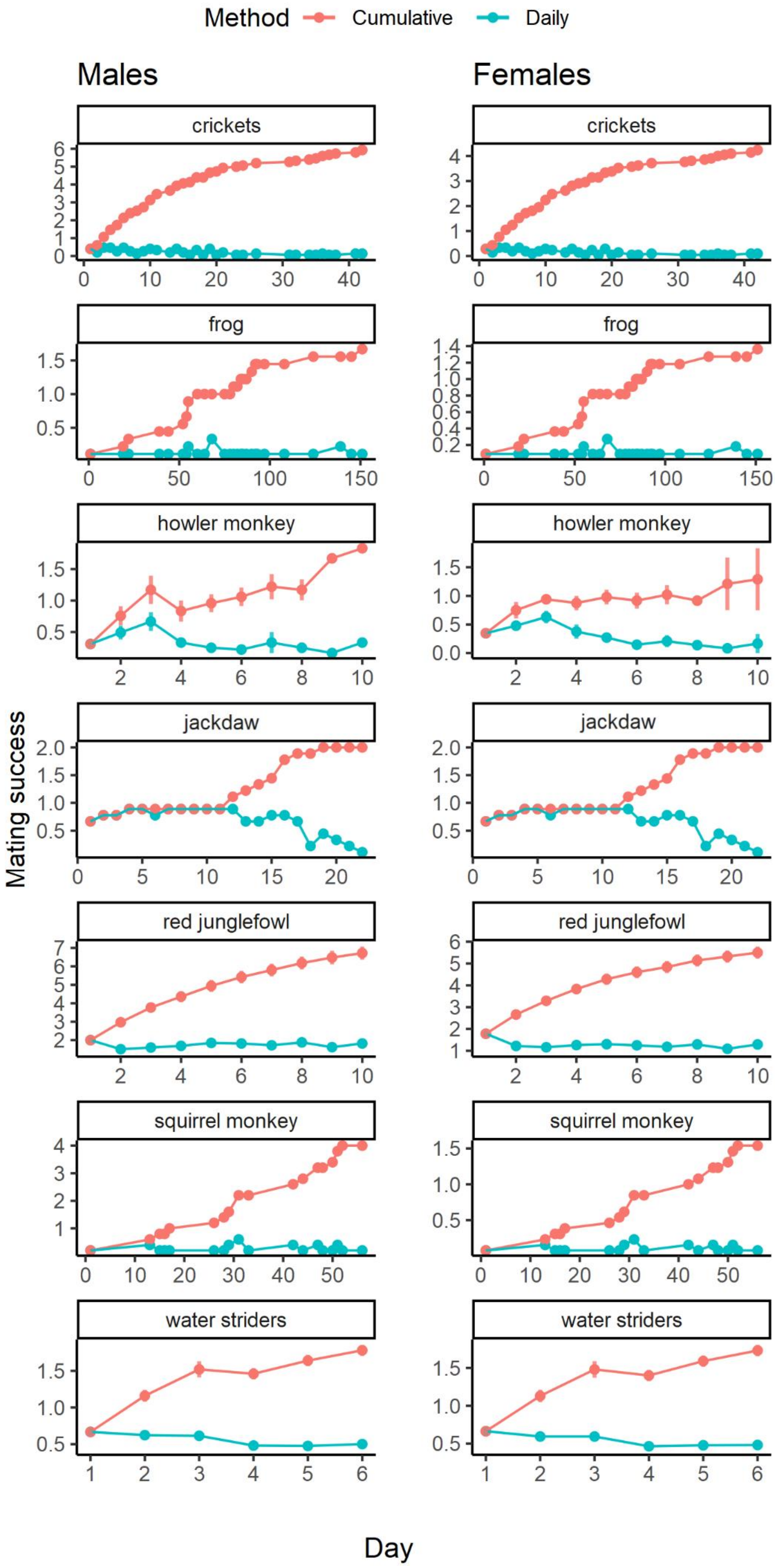
Method
Cumulative
Daily
Males
Females
crickets
frog
howler monkey
jackdaw
red junglefowl
squirrel monkey
water striders
Mating success
Day

Supplementary Figure 2 – Cumulative and daily mating success over time across multiple vertebrate and invertebrate species. Mean (s.e) mating success (i.e. number of unique mates) across individual males (left) and females (right) over time (days). Species include: Hawaiian swordtail crickets[2] (*Laupala cerasina*) *n* groups = 1, strawberry poison-dart frogs[5] (*Dendrobates pumilio*) *n* groups = 1, howler monkeys[4] (*Alouatta caraya*) *n* groups = 6, jackdaws[6] (*Corvus monedula*) *n* groups = 1, red junglefowl[7-10] (*Gallus gallus*) *n* groups = 20, squirrel monkeys[3] (*Saimiri oerstedi*) *n* groups = 1, and water striders[1] (*Aquarius remegis*) *n* groups = 40. Red points show values calculated cumulatively and represent the mean mating success in a given day taking into account mating success over all preceding time units. Green points show daily instantaneous values where mating success is assessed independently for each time unit (day). In species where multiple unique groups are studied (e.g., howler monkeys), the identity of groups measured on a given day may change, resulting in a decrease in mean cumulative mating success.

References

1. Sih, A., Montiglio, P.-O., Wey, T. W. & Fogarty, S. Altered physical and social conditions produce rapidly reversible mating systems in water striders. *Behav. Ecol.* **28**, 632–639 (2017).

2. Turnell, B. R. & Shaw, K. L. High opportunity for postcopulatory sexual selection under field conditions. *Evolution* **69**, 2094–2104 (2015).

3. Boinski, S. Mating patterns in squirrel monkeys (*Saimiri oerstedi*): implications for seasonal sexual dimorphism. *Behav. Ecol. Sociobiol.* **21**, 13–21 (1987).

4. Oklander, L. I., Kowalewski, M. & Corach, D. Male reproductive strategies in black and gold howler monkeys (*Alouatta caraya*). *Am. J. Primatol.* **76**, 43–55 (2014).

5. Pröhl, H. & Hödl, W. Parental investment, potential reproductive rates, and mating system in the strawberry dart-poison frog, *Dendrobates pumilio*. *Behav. Ecol. Sociobiol.* **46**, 215–220 (1999).

6. Gill, L. F., van Schaik, J., von Bayern, A. M. P. & Gahr, M. L. Genetic monogamy despite frequent extrapair copulations in “strictly monogamous” wild jackdaws. *Behav. Ecol.* **31**, 247–260 (2020).

7. McDonald, G. C., Spurgin, L. G., Fairfield, E. A., Richardson, D. S. & Pizzari, T. Pre- and postcopulatory sexual selection favor aggressive, young males in polyandrous groups of red junglefowl. *Evolution* **71**, 1653–1669 (2017).

8. McDonald, G. C., Spurgin, L. G., Fairfield, E. A., Richardson, D. S. & Pizzari, T. Differential female sociality is linked with the fine-scale structure of sexual interactions in replicate groups of red junglefowl, *Gallus gallus*. *Proc. Royal Soc. B* **286**, 20191734 (2019).

9. Carleial, R., McDonald, G. C. & Pizzari, T. Dynamic phenotypic correlates of social status and mating effort in male and female red junglefowl, *Gallus gallus*. *J. Evol. Biol.* **33**, 22–40 (2020).

10. Carleial, R. *et al.* Temporal dynamics of competitive fertilization in social groups of red junglefowl (*Gallus gallus*) shed new light on avian sperm competition. *Philos. Trans. R. Soc. B* **375**, 20200081 (2020).
