# Supplementary material for: Disentangling the causes of temporal variation in the opportunity for sexual selection
Source: Nat Commun. 2023 Feb 22;14:1006. doi: 10.1038/s41467-023-36536-7 (PMC9947164; doi:10.1038/s41467-023-36536-7)
Supplement: Supplementary file 3 — Reporting Summary [file 41467_2023_36536_MOESM3_ESM.pdf]

nature portfolio

Corresponding author(s): Rômulo Carleial
Grant C. McDonald

Last updated by author(s): Jan 31, 2023

Reporting Summary

Nature Portfolio wishes to improve the reproducibility of the work that we publish. This form provides structure for consistency and transparency in reporting. For further information on Nature Portfolio policies, see our Editorial Policies and the Editorial Policy Checklist.

Statistics

For all statistical analyses, confirm that the following items are present in the figure legend, table legend, main text, or Methods section.

n/a	Confirmed	
☐	☒	The exact sample size (*n*) for each experimental group/condition, given as a discrete number and unit of measurement
☐	☒	A statement on whether measurements were taken from distinct samples or whether the same sample was measured repeatedly
☐	☒	The statistical test(s) used AND whether they are one- or two-sided<br>*Only common tests should be described solely by name; describe more complex techniques in the Methods section.*
☐	☒	A description of all covariates tested
☐	☒	A description of any assumptions or corrections, such as tests of normality and adjustment for multiple comparisons
☐	☒	A full description of the statistical parameters including central tendency (e.g. means) or other basic estimates (e.g. regression coefficient) AND variation (e.g. standard deviation) or associated estimates of uncertainty (e.g. confidence intervals)
☐	☒	For null hypothesis testing, the test statistic (e.g. *F*, *t*, *r*) with confidence intervals, effect sizes, degrees of freedom and *P* value noted<br>*Give P values as exact values whenever suitable.*
☒	☐	For Bayesian analysis, information on the choice of priors and Markov chain Monte Carlo settings
☒	☐	For hierarchical and complex designs, identification of the appropriate level for tests and full reporting of outcomes
☒	☐	Estimates of effect sizes (e.g. Cohen's *d*, Pearson's *r*), indicating how they were calculated

Our web collection on statistics for biologists contains articles on many of the points above.

Software and code

Policy information about availability of computer code

Data collection	No software was used for data collection.
Data analysis	We used R version 3.6.2 for all analyses and the following packages:<br>"lme4 v1.1-30", "lmerTest v3.1-3"<br>Custom R scripts for random mating simulations and shuffling of the day order have been deposited in the figshare database under accession code https://doi.org/10.6084/m9.figshare.21902133.v1.

For manuscripts utilizing custom algorithms or software that are central to the research but not yet described in published literature, software must be made available to editors and reviewers. We strongly encourage code deposition in a community repository (e.g. GitHub). See the Nature Portfolio guidelines for submitting code & software for further information.

Data

Policy information about availability of data

All manuscripts must include a data availability statement. This statement should provide the following information, where applicable:

- Accession codes, unique identifiers, or web links for publicly available datasets
- A description of any restrictions on data availability
- For clinical datasets or third party data, please ensure that the statement adheres to our policy

The data generated in this study have been deposited in the figshare database under accession code https://doi.org/10.6084/m9.figshare.21902133.v1 91. Source

data are provided as a Source Data file.

Human research participants

Policy information about studies involving human research participants and Sex and Gender in Research.

Reporting on sex and gender	We did not use human datasets.
Population characteristics	We did not use human datasets.
Recruitment	We did not use human datasets.
Ethics oversight	We did not use human datasets.

Note that full information on the approval of the study protocol must also be provided in the manuscript.

Field-specific reporting

Please select the one below that is the best fit for your research. If you are not sure, read the appropriate sections before making your selection.

☐ Life sciences ☐ Behavioural & social sciences ☒ Ecological, evolutionary & environmental sciences

For a reference copy of the document with all sections, see nature.com/documents/nr-reporting-summary-flat.pdf

Ecological, evolutionary & environmental sciences study design

All studies must disclose on these points even when the disclosure is negative.

Study description

This study uses previously published mating data from the literature (60 datasets across 7 studies) from multiple animal species (n=7) to investigate temporal variation in opportunities for sexual selection. We calculated opportunities over successive days in two ways, (1) cumulatively, where estimates considered reproductive patterns from all preceding days; and (2) instantaneously, where estimates were considered independently on each day. We evaluated temporal trends in opportunities and the impact of cumulative versus daily instantaneous approaches for each species and sex separately. We used linear regression analyses for studies without replicate groups, and linear mixed-effects models (LMMs) including a random effect of group identity for studies with more than one replicate group. Finally, we assessed whether observed temporal trends in opportunities across males and females deviated from trends calculated from simulations assuming random mating for all species.

Research sample

We used seven published studies to calculate temporal changes in the opportunity for sexual selection:
Sih et al. 2017 - 24 groups of water striders (Aquarius remigis) containing 3 males and 3 females each were observed for 6 consecutive days.
Turnell & Shaw 2015 - A group of swordtail crickets (Laupala cerasina) comprising 20 males and 20 females was observed over 6 weeks. A total of 6 older females were substituted by 6 young females on the 5th day of the experiment. A total of 5 males and a single female died over the experiment and were replaced with new individuals.
Boinski 1987 - A single troupe of squirrel monkeys (Saimiri oerstedi) ranging from 38-45 individuals was observed for 17 to 21 days per month for 11 months. 10 males and 16 females were reproductively mature during observations.
Oklander et al. 2014 - 2 multi-male multi-female howler monkey (Alouatta caraya) groups were observed on 5 separated sampling periods lasting 2 weeks each. One group had 3 adult males and 4 adult females, whereas the other had 3 adult males and 2 adult females. Extra-group matings occurred in 2 sampling seasons.
Pröhl & Hödl 1999 - Strawberry dart-poison frogs (Dendrobates pumilio) were observed in the field for 143 consecutive days. 9 males and 11 females mated over the sampling period.
Gill et al. 2020 - 8 jackdaw (Corvus monedula) nest boxes were sampled over a period of 22 days using video recordings. 18 individuals mated and were properly identified.
McDonald et al. 2017 - Freely-mating groups (n=20) of red junglefowl (Gallus gallus) containing 10 males and 12 females each. Groups were observed 6h a day, and eggs were collected at the end of each day. Parentage analysis was performed for each fertilised egg.
Studies were chosen insofar as they contained behavioural data of freely mating groups that provided information on which males copulated with which females, required to calculate temporal patterns in opportunity for sexual selection over more than one time point. These datasets are meant to represent temporal variation in the opportunity for sexual selection across a subset of animal species.

Sampling strategy

We searched the literature for studies containing longitudinal mating data allowing for the calculation of variance in male and female mating success over successive days. Each dataset therefore required a minimum of two days of mating data. We used a search strategy aimed at identifying the widest range of species and populations as possible. The resulting sample size was chosen as it represents the maximum number of appropriate datasets which could be attained from relevant literature searches.

Data collection

We searched the literature for suitable studies. Search terms were tailored to locate studies with behavioural mating data or reporting sexual selection metrics requiring the appropriate raw mating data. The first search used the TOPIC field and contained the following search terms (“Bateman* gradient*”) OR (“Bateman* slope*”) OR (“Bateman* principle*”) OR (“opportunity* for selection”) OR (“opportunity* for sexual selection”) AND (“Sexual selection”). This resulted in 35 records. The second search included

	the TOPIC terms (sexual network* OR social network*) AND (sexual selection OR mating system). This search returned 234 records. The third search contained the TOPIC terms (mating* or copulat*) AND (behavio*) AND (observ*), restricting our search to the journals Animal Behavior, Behavioral Ecology, and Behavioral Ecology and Sociobiology. This returned 100 records. All searched spanned the following Web of Science indexes: SCI-EXPANDED, SSCI, A&HCI, CPCI-S, CPCI-SSH, BKCI-S, BKCI-SSH, ESCI, CCR-EXPANDED, IC. Original data are available in public online databases, or tabulated in the original papers. Data for the present study was collated by Grant C. McDonald.
Timing and spatial scale	Literature searches were originally performed on February 14, 2017 during a different study (McDonald and Pizzari 2017). To obtain mating data published after the study by McDonald and Pizzari 2017 we repeated similar updated versions of the searches on Web of Science dated spanning from the time of the original search (14th February 2017) until the 19th March 2021 (i.e. searches were limited to 2017-2021). Archived data used in this study comprised 7 locations across 5 countries: California, USA; Hawaii, USA; Oxford, UK; Chaco Province, Argentina; Hitoy Cerere, Costa Rica; Bavaria, Germany; Peninsula de Osa, Costa Rica.
Data exclusions	Studies with group sizes of only two males or females, or where individuals were not allowed to freely interact and copulate (i.e., repeated experimental pairs) were not considered. Similarly, studies relying solely on molecular parentage to infer mating data with no behavioural mating observations were not included. This returned a total of seven studies with suitable data.
Reproducibility	All main results of this paper can be reproduced with the data archived along with this paper, together with previously published datasets. R codes, with worked examples, have also been made available along with the present study. Data and R scripts have been deposited in figshare under accession code https://doi.org/10.6084/m9.figshare.21902133.v1.
Randomization	No experiments were conducted during this study, as we only used published datasets. Therefore, randomizations are not relevant to the present study.
Blinding	Blinding was not relevant to this study because investigators simply collected data that had been previously published.

Did the study involve field work? ☐ Yes ☒ No

Reporting for specific materials, systems and methods

We require information from authors about some types of materials, experimental systems and methods used in many studies. Here, indicate whether each material, system or method listed is relevant to your study. If you are not sure if a list item applies to your research, read the appropriate section before selecting a response.

Materials & experimental systems

n/a	Involved in the study
☒	☐ Antibodies
☒	☐ Eukaryotic cell lines
☒	☐ Palaeontology and archaeology
☒	☐ Animals and other organisms
☒	☐ Clinical data
☒	☐ Dual use research of concern

Methods

n/a	Involved in the study
☒	☐ ChIP-seq
☒	☐ Flow cytometry
☒	☐ MRI-based neuroimaging
